# Green Fabrication of Phosphocreatine Intercalated Layered Double Hydroxides for Highly Efficient Flame-Retardant Epoxy Nanocomposites

**DOI:** 10.3390/polym18091118

**Published:** 2026-04-30

**Authors:** Xuqi Yang, Shuyi Zhang, Marjan Entezar Shabestari, Abbas Mohammadi, Bahareh Hoomehr, Ehsan Naderi Kalali, Saeid Lotfian

**Affiliations:** 1 Department of Safety Engineering, Faculty of Geoscience and Engineering, Southwest Jiaotong University, 111 2nd Ring Rd North Section 1, Jinniu District, Chengdu 610032, China; yangxuqi@my.swjtu.edu.cn (X.Y.); marjanentezar8@gmail.com (M.E.S.); 2 Department of Mechanical Engineering, University of Michigan-Ann Arbour, 503 Thompson Street, Ann Arbour, MI 48109, USA; shuyz@umich.edu; 3 Department of Chemistry, University of Isfahan, Isfahan 81746-73441, Iran; a.mohammadi@sci.ui.ac.ir; 4 Department of Materials Science and Engineering, School of Engineering, Shiraz University, Shiraz 84334-71964, Iran; bahar.hoomehr@saadi.shirazu.ac.ir; 5 Department of Naval Architecture, Ocean and Marine Engineering, University of Strathclyde, Glasgow G4 0LZ, UK

**Keywords:** epoxy nanocomposites, phosphocreatine, layered double hydroxides, flame retardancy, thermal properties

## Abstract

We co-modified layered double hydroxide (LDH) in water using phosphocreatine (PC) and dodecylphosphoric acid (DPA) to obtain a highly dispersible LDH. Embedding this LDH in epoxy enabled V-0 at 7 wt% and lowered HRR, THR and TSP, attributed to a dense char and PC-DPA synergy. SEM, WAXS, and TGA characterised the structure and thermal behaviour of the functionalised LDHs. These modified LDHs were then loaded into the epoxy resin (EP) to develop flame-retardant nanocomposites. Compared to unmodified LDH (NO_3_-LDH) and PC-modified LDH (PC-LDH), PC-DPA-LDH showed superior dispersion and compatibility within the epoxy matrix. As a result, PC-DPA-LDH/EP achieved a UL-94 V-0 rating at only 7 wt% loading, while NO_3_-LDH/EP had no rating, and PC-LDH/EP reached only V-2. Moreover, PC-DPA-LDH/EP demonstrated significant decreases in peak heat release rate (46.4%), total heat release (34.5%), and total smoke production (59.7%) compared with neat EP. These improvements were attributed to the synergistic flame-retardant effects of PC and DPA, as well as to the formation of a compact char layer that effectively insulated the underlying material and suppressed volatile emissions. This work highlights the potential of bio-based, aqueous-synthesised nanohybrids for high-efficiency, eco-friendly flame-retardant epoxy systems.

## 1. Introduction

Epoxy resins (EPs) have attracted significant attention in recent decades due to their ease of processing, low cost, excellent adhesive properties, superior mechanical properties, and solvent resistance [1,2,3,4,5]. As a result, EPs are widely used as composite matrices in electronics and electrical devices, aeronautics, high-speed trains, etc., particularly where high flame-retardant properties are necessary [6,7]. However, their tendency to catch fire is a major drawback [8,9,10]. For many years, epoxy resins were made flame-resistant using brominated or chlorinated additives [11,12]. While effective, these additives raised persistent environmental and health concerns. Consequently, in recent years, researchers have focused on developing safer, halogen-free flame retardants to replace traditional halogen-based chemicals [13,14,15].

Conventional phosphorus (P), silicon (Si) or boron (B) containing additives often need high loadings. In particular, phosphorus- and nitrogen-containing flame retardants have been widely investigated for epoxy resins because they can promote char formation and improve flame retardancy of the polymeric matrices. However, effective fire protection in such systems often still requires relatively high additive loading or careful molecular design to avoid compromising the mechanical properties, thermal stability, or processability of the epoxy matrix. As an example, epoxy-containing phosphazene systems have shown improved fire resistance. Still, in some cases, self-extinguishing behaviour was achieved only at very high phosphazene contents of up to 75 wt%. In contrast, more recent imidazole-containing phosphorus-nitrogen flame retardants demonstrate that effective flame retardancy can be achieved at much lower loadings, with UL-94 V-0 reached at only 3.0 wt%, when the molecular structure is carefully optimised [16,17]. Nanoscale fillers, by contrast, can deliver comparable or better fire performance at lower dosages, mitigating property penalties [18,19,20,21]. Nanocomposite approaches offer a way around this issue. Even at low additive levels, they can dramatically enhance flame retardancy [22]. Nanotechnology has proven highly effective at enhancing the flame-retardant properties of polymers, offering superior suppression of heat release rates compared with traditional flame retardants [23]. For example, adding only 10% clay to polypropylene reduced the peak heat release rate by 70%, as measured by a cone calorimeter [24].

In addition to improving fire resistance, nano-additives also enhance the mechanical properties of materials, a property that most conventional flame retardants cannot achieve [25]. One such nano-additive is layered double hydroxide (LDH), a host framework with confined species constructed from positively charged hydroxide sheets, with interlayer spaces occupied by charge-compensating anions and water. Changing the divalent/trivalent metal ratio (e.g., Mg-Al, Zn-Al) tunes chemistry and spacing [26,27]. Since 2001, LDH/polymer composites have attracted increasing interest, and LDHs have been successfully incorporated into various polymer matrices, such as polypropylene, rubber, polyamide, and polylactic acid [28].

To increase fire resistance in industrial applications, LDHs are often combined with phosphorus-containing chemicals, resulting in a synergistic effect [29]. However, because pristine LDH stacks disperse poorly in polymers, intercalating anionic surfactants is routinely used to widen galleries and improve compatibility [30]. Many types of anionic surfactants have been employed for this purpose, including fatty acid salts, organosulfonates, and organophosphates, as these intercalate into LDH layers and aid dispersion. These modifications improve the dispersion of LDHs, thereby enhancing the properties of the resulting polymer composites [31].

In earlier work, we found that modifying LDH can reduce flammability, but a single modifier did not sufficiently expand the LDH galleries to achieve optimal nanocomposite dispersion. Accordingly, we adopt a dual-modifier strategy: PC for char promotion and DPA for gallery widening, to target both FR and mechanical properties. We synthesised phosphocreatine (PC) as the primary modifier for LDH, as it contains nitrogen units that promote char residue formation. Sodium dodecylphosphoric acid (DPA) was used as a secondary co-modifier to further expand the interlayer distance of LDH, thereby improving its dispersion in the polymer matrix. In contrast, its phosphorus group can form phosphoric acid, phosphorus oxides, and related compounds during combustion. These gases can act as flame inhibitors in the gas phase by hindering the free-radical reactions necessary for combustion. Therefore, this work introduces a green, aqueous co-modification strategy for LDH that integrates interlayer expansion, improved nanofiller dispersion, and synergistic flame-retardant action within a single nanohybrid system. By scavenging these free radicals, DPA effectively slows combustion and reduces flame intensity. The prepared functionalised LDHs were analysed via scanning electron microscopy (SEM), wide-angle X-ray scattering (WAXS), and thermogravimetric analysis (TGA). Finally, the modified LDHs were incorporated into epoxy resins to prepare a series of flame-retardant epoxy composites, which were then systematically evaluated for Surface texture, thermal stability, flame-retardant performance, and mechanical properties.

## 2. Materials and Methods

### 2.1. Materials

Mg(NO_3_)_2_·6H_2_O (purity ≥ 98%), Al(NO_3_)_3_·9H_2_O (purity ≥ 98%), sodium hydroxide, creatine monohydrate, phosphoric acid and sodium dodecyl phosphoric acid (DPA) (purity ≥ 97%) were obtained from TCI Chemicals Company (Tokyo, Japan), 4,4′-Diaminodiphenylmethane (DDM, purity ≥ 99%) was purchased from Shanghai Aladdin Biochemical Technology Co., Ltd. (Shanghai, China), and used without further purification. Epoxy resin (E-44, epoxy equivalent weight: ~0.44–0.48 mol/100 g, dynamic viscosity: ~11,000–15,000 mPa·s at 25 °C) was supplied by Nantong Xingchen Co., Ltd. (Nantong, China).

### 2.2. Synthesis of Phosphocreatine

**Figure 1:**
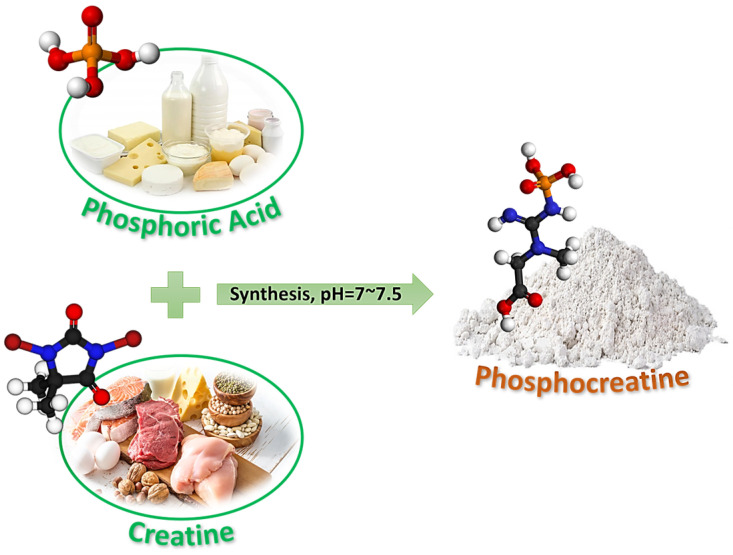
Synthesis procedure of phosphocreatine.

Phosphocreatine is a molecule that can be synthesised in the laboratory by reacting creatine with phosphoric acid (Figure 1). In a typical preparation procedure [32], the desired quantity of creatine monohydrate was added to a beaker. Then, the solution was stirred with a magnetic stir bar, and the creatine was dissolved in water or a buffered solution to form a homogeneous solution, with the concentration adjusted to ensure complete dissolution. For this work, the creatine phosphate concentration is adjusted to match that of Al(NO_3_)_3_·9H_2_O, which is 0.1 M.

The pH value of the solution was controlled between 7.0 and 7.5. In another beaker, a solution of phosphoric acid was prepared. The solution pH was adjusted from 1.5 to 2.0. While continuously stirring, the phosphate solution was added dropwise to the creatine solution. The solution pH was checked periodically and adjusted with sodium hydroxide or hydrochloric acid to maintain a pH of approximately 6.5–7.5. The mixture was then agitated at room temperature for several hours until the reaction was complete. The product mixture was filtered to remove solid impurities, and the filtrate was recovered by lyophilisation or solvent evaporation under reduced pressure.

### 2.3. Preparation of Functionalised LDHs

**Figure 2:**
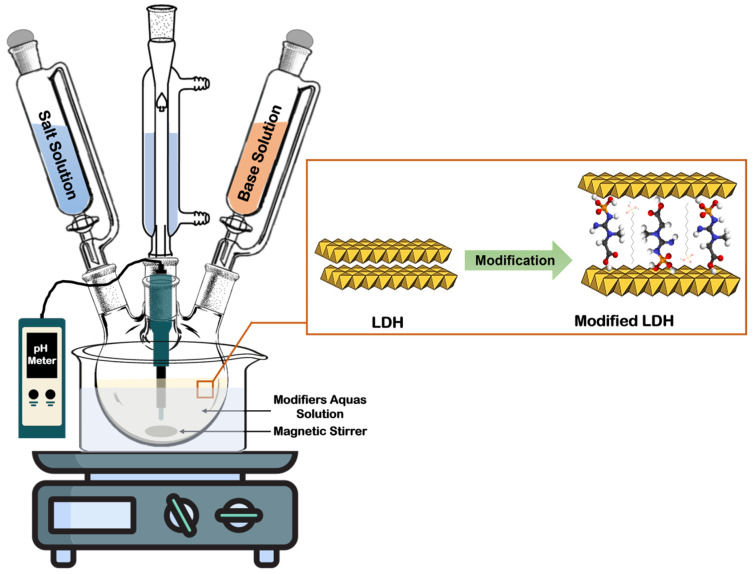
Schematic diagram of LDH synthesis and functionalisation via a one-step synthesis method.

Phosphocreatine (0.05 mol) and dodecyl phosphoric acid (0.05 mol) were added to a 1000 mL three-neck round-bottom flask fitted with an isoboric funnel, a reflux apparatus, and a pH meter. The mixture was stirred with 250 mL of deionised water until fully dissolved. Next, an aqueous solution containing Mg(NO_3_)_2_·6H_2_O (0.2 mol) and Al(NO_3_)_3_·9H_2_O (0.1 mol) in 250 mL of deionised water was gradually introduced into the flask. The pH was controlled at 10 ± 0.5 throughout the synthesis using 1 M NaOH. The obtained slurry was stirred continuously for 30 min, followed by ageing at 75 °C for 18 h. Afterwards, the mixture was filtered and thoroughly washed with deionised water until the pH reached 7. The filtered cake was dried in an oven at 80 °C until constant weight, yielding PC-DPA-LDH. Additionally, unmodified NO_3_-LDH and PC-LDH were synthesised via the same method. The synthesis routes are shown in Figure 2.

### 2.4. Preparation of LDH/Epoxy Nanocomposites

Pristine LDH (NO_3_-LDH) and functionalised LDH (PC-LDH and PC-DPA-LDH) were used to prepare LDH/epoxy nanocomposites at a constant weight fraction of 7 wt%. A multistep mixing method was employed to achieve the highest possible dispersion level. First, LDH or functionalised LDH was dispersed in the epoxy matrix via a three-roll mill (Guangdong AISRY Instrument Technology Co., Ltd., Guangzhou, China) for 20 min. In the next step, the mixture was diluted with acetone and ultrasonicated for 20 min at 60 °C, after which acetone was removed under vacuum at 110 °C, leaving a viscous mixture that facilitated the reaction between the amino groups in creatine and the epoxy chains. The mixture was then cooled to 65 °C, DDM was added, and the mixture was stirred for 15 min until DDM dissolved completely. The mixture was placed under vacuum at 70 °C for 5 min to remove trapped air before being poured into preheated silicone-rubber moulds. The curing process was conducted at 100 °C for 2 h, followed by 150 °C for 2 h. Following the above process, NO_3_-LDH/EP, PC-LDH/EP, and PC-DPA-LDH/EP were successfully prepared.

### 2.5. Characterisation

Fourier transform infrared (FTIR) spectra were collected using a Nicolet iS50 FTIR spectrometer (Thermo Fisher Scientific, Waltham, MA, USA) over the wavenumber range of 4000–500 cm^−1^, with 16 scans per spectrum at a resolution of 4 cm^−1^.

The Wide-Angle X-ray Scattering (WAXS) patterns of the samples were recorded on an X-ray diffractometer (Empyrean, Beijing, China) using Cu Kα radiation at 60 kV.

SEM was used to examine the morphologies of the LDHs before and after modification with a JSM-7800F Prime microscope (JEOL Ltd., Tokyo, Japan), equipped with an OXFORD X-Max 80 detector (High Wycombe, UK). The surface and cross-sectional morphologies of the char residues after cone calorimeter testing were further observed using a Zeiss EVO MA15 scanning electron microscope (ZEISS Group, Oberkochen, Germany). All the samples were sputter-coated with platinum under vacuum before observation.

TEM analysis was conducted using a JEOL transmission electron microscope (JEOL Co., Ltd., Tokyo, Japan) to examine the morphology and microstructure of the composites at high magnification. The samples were prepared using a Leica ultramicrotome to obtain ultrathin sections approximately 80 nm thick. TEM observations were carried out in bright-field mode at an accelerating voltage of 200 kV.

Cone calorimeter tests were carried out in accordance with ISO 5660-1 [33] using a cone calorimeter (Fire Testing Technology (FTT) Ltd., East Grinstead, UK). Square specimens with dimensions of 100 mm × 100 mm × 4 mm were wrapped in aluminium foil and mounted in a holder without a grid. The samples were exposed to an external heat flux of 50 kW/m^2^ to simulate a medium fire scenario. All cone calorimeter measurements were repeated at least twice, and the variation in major fire parameters, including pHRR, THR, and TSP, was within ±5%, indicating good reproducibility of the experimental results.

The limiting oxygen index (LOI) was measured using an HC-2C oxygen index meter (Nanjing Shangyuan Analytical Instrument Co., Ltd., Nanjing, China) in accordance with ASTM D2863-2012 [34]. The specimen dimensions were 130.0 mm × 6.5 mm × 3.2 mm.

The UL-94 vertical burning test was conducted using a CFZ-3 instrument (Nanjing Shangyuan Analytical Instrument Co., Ltd., Nanjing, China) in accordance with ASTM D3801-2010 [35], with specimen dimensions of 130.0 mm × 12.7 mm × 3.2 mm.

Thermogravimetric analysis (TGA) was performed using a thermal analyser (Netzsch TG 209 F1, Selb, Germany) with a heating rate of 10 °C/min from 50 °C to 800 °C under nitrogen flow. A linear combination of the TG curves for neat EP and LDH was used to calculate the theoretical TG curve. The formula is as follows:

W*_th_*(T)*_LDH/EP_* = x × W_exp_(T)*_EP_* + y × W_exp_(T)*_LDH_*, x + y = 1 (1)

where W_exp_(T)*_EP_* represents the experimental TG curve of pure EP; W_exp_(T)*_LDH_* represents the experimental TG curve of LDH; and x and y represent the weight fractions of EP and LDH in the composites, respectively.

Tensile tests were performed on dumbbell-shaped samples (75 mm × 10 mm × 2 mm) according to ISO 527-2 [36] using an Instron 5966 universal testing machine, (Norwood, MA, USA) at a test speed of 1 mm/min.

Charpy impact tests were conducted on unnotched specimens using a cantilever beam impact tester (XC-22Z, Chengde, China). The specimens were rectangular, measuring 50 mm × 6 mm × 4 mm, and prepared in accordance with ASTM D256-04 [37].

## 3. Results and Discussion

### 3.1. Characterisation of the Functionalised LDH

**Figure 3:**
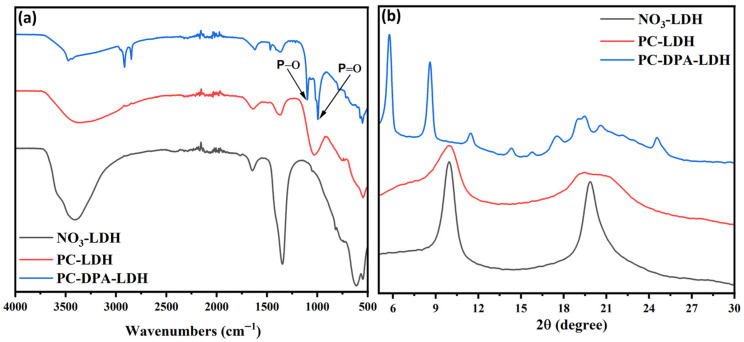
(**a**) FTIR spectra of NO_3_-LDH, PC-LDH, and PC-DPA-LDH; (**b**) WAXS patterns of NO_3_-LDH, PC-LDH, and PC-DPA-LDH.

The FTIR spectra of NO_3_-LDH, PC-LDH, and PC-DPA-LDH are shown in Figure 3a. The spectrum of unmodified NO_3_-LDH exhibited typical absorption bands, including a broad peak at approximately 3450 cm^−1^ associated with O-H stretching vibrations and a band near 1630 cm^−1^ corresponding to H-O-H bending of interlayer water. The strong peak at approximately 1385 cm^−1^ is assigned to the symmetric stretching of nitrate ions, confirming their presence in the interlayer region. After phosphocreatine modification, the nitrate peak intensity decreased significantly. At the same time, new absorption bands appeared between 1200 and 900 cm^−1^. In particular, the band near 1250 cm^−1^ is assigned to P=O stretching. Whereas, the bands in the ~1050–970 cm^−1^ region are attributed to P–O–C stretching vibrations, indicating successful incorporation of phosphocreatine. Although phosphocreatine was not isolated for separate characterisation, its successful formation and intercalation are supported by the appearance of characteristic phosphate-related bands in the FTIR spectra of the modified LDHs. In the PC-DPA-LDH spectrum, the distinct peaks at approximately 2950 and 2850 cm^−1^, corresponding to the C-H stretching of alkyl chains, confirmed the presence of dodecyl phosphoric acid. The increased complexity and intensity of the phosphate- and alkyl-related bands further verify the effective co-modification of LDH by both phosphocreatine and dodecyl phosphoric acid, accompanied by the near-complete removal of nitrate species.

WAXS analysis further confirms that co-modification dramatically expands the LDH gallery (Figure 3b). Compared to NO_3_-LDH (d-spacing ~0.8 nm at 2θ ≈ 9.7°), the PC-DPA-LDH shows its (003) peak at a much lower angle, corresponding to ~1.7 nm gallery height. This roughly doubles the interlayer spacing of NO_3_-LDH. The substantial increase reflects the incorporation of the bulky DPA chains; for perspective, a DPA molecule (~2 nm) far exceeds the thickness of a nitrate anion (~0.1 nm) [21].

**Figure 4:**
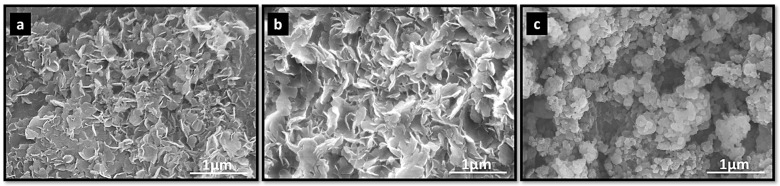
SEM images of (**a**) NO_3_-LDH, (**b**) PC-LDH and (**c**) PC-DPA-LDH.

Figure 4 shows SEM micrographs of the morphological features of NO_3_-LDH and the modified LDHs. NO_3_-LDH (Figure 4a) exhibits a plate-like morphology composed of primary particles with microscopically smooth surfaces. The sharp edges observed in many particles may indicate incomplete crystal growth, leading to a lack of well-defined particle shape. The modified LDHs (Figure 4b,c) show morphological features similar to those of NO_3_-LDH and retain the typical layered structure, indicating that anion intercalation did not change the two-dimensional morphology of the LDH. In addition, PC-DPA-LDH exhibited a relatively looser and less aggregated lamellar morphology, which can be attributed to the intercalation of PC and DPA molecules that increased the interlayer spacing and weakened the stacking of LDH platelets.

### 3.2. Structural Characterisation of LDH/EP Nanocomposites

X-ray diffraction patterns (WAXS) provide insight into the dispersion of LDH in epoxy, as shown in Figure S1 (Supplementary Information). Neat epoxy shows only a broad halo (~2θ = 19°) from its amorphous structure. By contrast, the NO_3_-LDH/epoxy composite exhibits a sharp diffraction peak at ~10° (d~0.8 nm), signifying that the unmodified LDH remains in ordered and layered form within the polymer. PC-LDH/EP yields a similar 10° peak, but much weaker, suggesting that fewer LDH layers remain stacked (most are partially delaminated). Notably, PC-DPA-LDH/EP shows no LDH peak in the 2θ = 5–10° range, which suggests that the LDH is almost completely exfoliated in the cured epoxy. This is consistent with TEM observations of a nearly homogeneous LDH dispersion.

**Figure 5:**
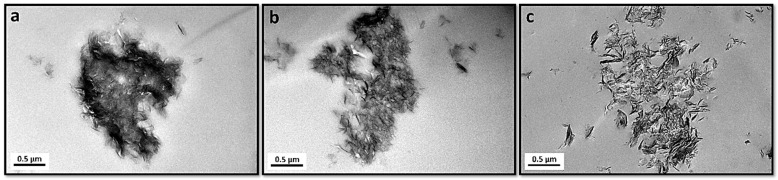
TEM images of (**a**) NO_3_-LDH/EP, (**b**) PC-LDH/EP, and (**c**) PC-DPA-LDH/EP nanocomposites.

TEM analysis was conducted to provide additional insights and validate the findings from the WAXS analysis while also directly observing the dispersion state of LDH within the epoxy matrix. Figure 5 presents several TEM images.

The TEM image of the NO_3_-LDH/EP composite (Figure 5a) revealed the presence of large NO_3_-LDH aggregates, with an average size exceeding 700 nm. Similarly, in the TEM measurements of the PC-LDH/EP composite (Figure 5b), the ordered structure of LDH was clearly visible. This observation aligns with the WAXS data, in which the (003) reflection peak remained evident for both NO_3_-LDH/EP and PC-LDH/EP. In contrast, the dispersion state of PC-DPA-LDH/EP was markedly different. The TEM image of PC-DPA-LDH/EP (Figure 5c) demonstrated a significantly improved dispersion of LDH within the matrix. This image depicted a homogeneously disordered microstructure, which corresponded well with the WAXS observations.

### 3.3. Thermal Stability

The thermal stability of the functionalised LDH and LDH-based epoxy nanocomposites was analysed, and the key findings are summarised in Table 1.

**Figure 6:**
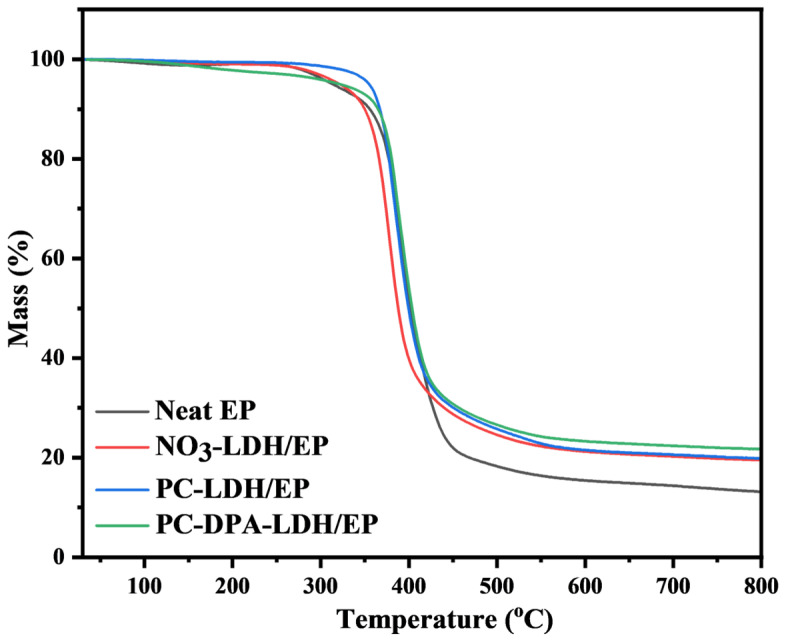
TGA curves of pure EP and epoxy nanocomposites under N_2_ at a heating rate of 10 °C/min.

Figure 6 shows the thermal stability of EP and its nanocomposites. Pure EP exhibited a main degradation stage in the 380–500 °C range, attributed to the decomposition of the crosslinked polymer network. All epoxy composites showed a similar single-step degradation behaviour. The incorporation of the functionalised LDHs increased the char residue at 800 °C. While pure EP retained 13.1% of the residue at 800 °C, the PC-LDH/EP and PC-DPA-LDH/EP composites gained 19.8% and 21.7% of the residue, respectively. Notably, the experimental char residues for PC-LDH/EP and PC-DPA-LDH/EP exceeded the calculated values, indicating a synergistic effect among the modifiers.

**Figure 7:**
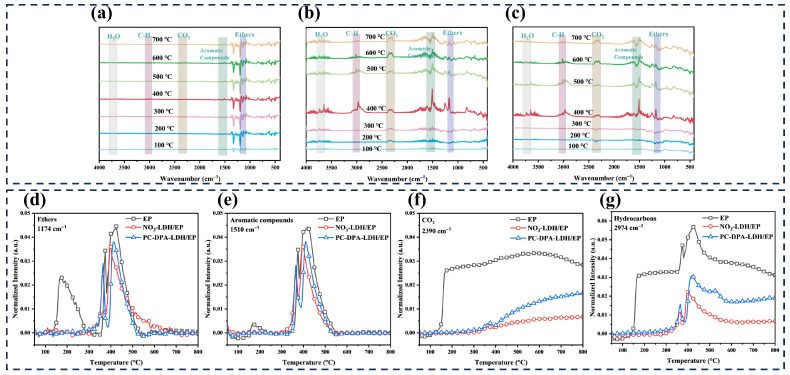
FTIR spectra of the pyrolysis gas products of (**a**) EP, (**b**) NO_3_-LDH/EP and (**c**) PC-DPA-LDH/EP at different temperatures; temperature–absorbance curves of (**d**) ethers, (**e**) aromatic compounds, (**f**) CO_2_ and (**g**) hydrocarbons of EP composites.

TG-IR analysis was further used to investigate the volatile products released during thermal degradation. In the 3D FTIR spectra, the intensity of the gaseous products can be compared along the *Z*-axis and in the colour distribution, which represents absorbance. Compared with pure EP (Figure S2a), all epoxy composites showed markedly lower absorbance intensities, indicating a reduced release of volatile pyrolysis products. This result suggests that the incorporation of inorganic flame retardants effectively suppressed the evolution of gaseous products during EP pyrolysis. The 3D FTIR spectra of the gas-phase pyrolysis products at different temperatures were further analysed, and the corresponding spectra of the epoxy composites are shown in Figure 7a–c. The main gaseous products released from EP and its composites include ethers (1150–1300 cm^−1^), aromatic compounds (1400–1600 cm^−1^), carbon dioxide (2390 cm^−1^), hydrocarbons (2900–3100 cm^−1^), and water vapour (3600–3750 cm^−1^), which are typical pyrolysis products of epoxy resin. Among them, pure EP (Figure 7a) exhibited relatively strong absorption signals in the range of 400–1600 cm^−1^, corresponding mainly to ethers and aromatic compounds, whereas the signals in the range of 1600–4000 cm^−1^ were comparatively weaker. In contrast, NO_3_-LDH/EP (Figure 7b) and PC-DPA-LDH/EP (Figure 7c) displayed a more uniform distribution of absorbance over the full wavenumber range. Figure 7d–g presents the temperature-dependent absorbance curves of characteristic gaseous products, including ethers (1176 cm^−1^), aromatic compounds (1510 cm^−1^), CO_2_ (2390 cm^−1^), and hydrocarbons (2974 cm^−1^). The absorbance intensities of ethers and aromatic compounds for pure EP were close to those of the other epoxy composites. However, the absorbance intensities of CO_2_ and hydrocarbons for pure EP were significantly higher than those of the flame-retardant epoxy composites. This finding indicates that fewer combustible pyrolysis products were released into the gas phase in the presence of LDH-based flame retardants, which can be attributed to the improved barrier effect of the char layer. Therefore, the TG-IR results demonstrate that the inorganic flame retardants inhibited the transfer of volatile degradation products into the gas phase and confirmed their synergistic flame-retardant action in both the condensed and gas phases.

### 3.4. Flammability Test

The LOI values and UL-94 classifications of pure epoxy and its composites are summarised in Table 2. Pure epoxy exhibited an LOI value of 25.0% and did not achieve any rating in the UL-94 vertical burning test. The addition of NO_3_-LDH increased the LOI value to 25.5%, but it still did not pass the UL-94 test. The incorporation of PC-LDH slightly improved the LOI value, but neither composite achieved a UL-94 V-0 rating. However, adding PC-DPA-LDH to the epoxy significantly enhanced its fire resistance, resulting in a UL-94 V-0 rating in the vertical burning test.

**Figure 8:**
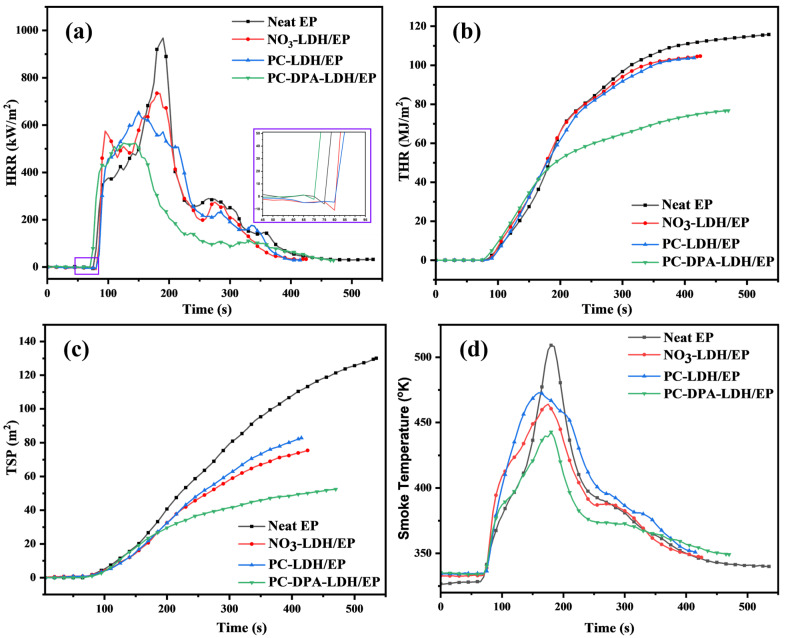
(**a**) Heat release rate, (**b**) total heat release, (**c**) total smoke production rate and (**d**) smoke temperature versus time curves of epoxy and its composites from cone calorimeter tests.

Cone calorimetry is widely used to evaluate the combustion behaviour of polymeric materials. Figure 8 presents the heat release rate (HRR), total heat release (THR), total smoke production (TSP), and smoke temperature curves of pure EP and its nanocomposites as a function of time. Other key parameters obtained from the cone calorimeter tests, including the time to ignition (TTI), peak heat release rate (pHRR), fire growth rate index (FIGRA), average HRR (Ave. HRR), and char residue, are summarised in Table 3. Compared with pure EP, the peak HRRs of the NO_3_-LDH/EP and PC-LDH/EP nanocomposites decreased from 970 kW/m^2^ to 733 kW/m^2^ (24% reduction) and to 651 kW/m^2^ (33% reduction), respectively. In the case of the PC-DPA-LDH/EP composites, as shown in Figure 8a, PC-DPA-LDH/EP ignited rapidly because of the early initial degradation stage after ignition. Compared with that of pure epoxy, the pHRR of PC-DPA-LDH/EP decreased by 46.4% to 520 kW/m^2^. A similar trend was observed for the average HRR values of pure EP and the epoxy nanocomposites, as listed in Table 3. Pure EP exhibited an average HRR of 361 kW/m^2^, while the values for NO_3_-LDH/EP and PC-LDH/EP decreased to 341 and 285 kW/m^2^, respectively. Among all samples, PC-DPA-LDH/EP showed the lowest average HRR of 236 kW/m^2^, corresponding to a 35% reduction compared with pure EP.

The improved fire performance of PC-DPA-LDH/EP can be attributed to better dispersion of the LDH layers within the epoxy matrix, resulting from the increased interlayer spacing of PC-DPA-LDH, as well as to enhanced char formation during combustion. In particular, the presence of PC and DPA species played a key role in enhancing the flame-retardant performance of PC-DPA-LDH. As shown in Figure 8b, pure EP released a total heat of 116 MJ/m^2^ at the end of burning, while NO_3_-LDH/EP released a similar amount of heat (105 MJ/m^2^). In contrast, the PC-DPA-LDH/EP nanocomposite released only 76 MJ/m^2^, corresponding to a 34.5% reduction in THR compared with neat EP. The significant reduction in THR meant that more organic structures in the epoxy resin participated in the carbonisation process and remained in the condensed phase rather than becoming “fuel” in the gas phase. This was also evidenced by the increased char residues (as listed in Table 3). With respect to the TSP, that of pure EP remains the highest among all the samples. As shown in Figure 8c, all the samples containing LDH had relatively lower TSPs than did the pure EP samples, with PC-DPA-LDH/EP showing a substantial reduction of 59.7% in TSP compared with neat EP. The decrease in smoke formation in the epoxy composites was probably due to the reduced amount of epoxy converted into organic volatiles, since organic volatiles are the major source of smoke particles [30,31].

Regarding the smoke temperature (Figure 8d), the neat EP exhibited the highest peak at 520 °C, indicating intense combustion. The incorporation of LDH-based additives reduced this peak, with NO_3_-LDH/EP and PC-LDH/EP reaching 485 °C and 465 °C, respectively. The lowest temperature at 440 °C was observed for PC-DPA-LDH/EP, indicating a decreased combustion intensity due to enhanced char formation and thermal barrier effects.

**Figure 9:**
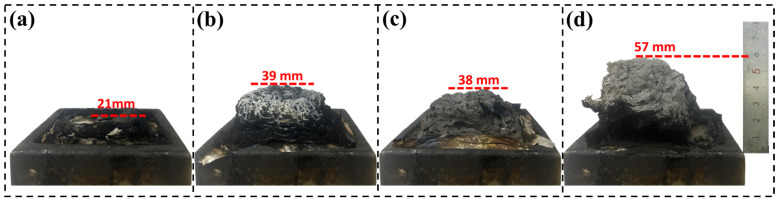
Digital photos of char residues after cone calorimeter testing: (**a**) Neat EP, (**b**) NO_3_-LDH/EP, (**c**) PC-LDH/EP, (**d**) PC-DPA-LDH/EP.

Cone calorimeter tests revealed that the mass loss and char yield of the PC-LDH/EP and PC-DPA-LDH/EP composites were significantly lower than those of the other samples, suggesting that their fire resistance is due mainly to a condensed-phase mechanism. A higher char yield indicates less epoxy breakdown into flammable volatiles. A dense, continuous char layer formed on the surface, acting as a thermal barrier that reduced heat transfer, limited combustible gas release, and blocked oxygen from reaching the combustion zone. This barrier effect helped suppress the flames. To further investigate the condensed-phase flame-retardant mechanism, digital images of the char residues are shown in Figure 9.

The neat EP formed a thin, compact char layer measuring 21 mm in height, indicating poor thermal protection. In contrast, the addition of LDH significantly enhanced char expansion. PC-DPA-LDH/EP produced the most expanded and voluminous char at 57 mm, followed by NO_3_-LDH/EP and PC-LDH/EP, demonstrating better insulation and intumescent behaviour.

**Figure 10:**
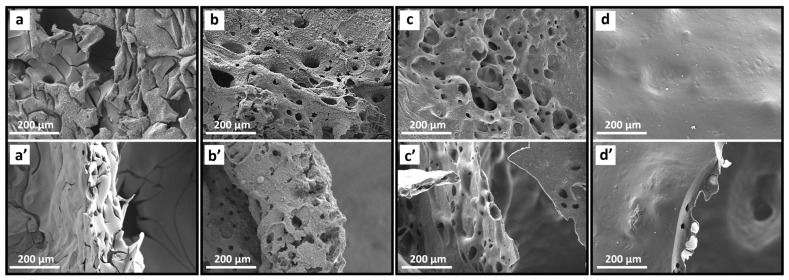
SEM images of char residues after cone calorimeter testing: (**a**,**a’**) Neat EP, (**b**,**b’**) NO_3_-LDH/EP, (**c**,**c’**) PC-LDH/EP, (**d**,**d’**) PC-DPA-LDH/EP.

The SEM images (Figure 10) further support these findings. Neat EP exhibited a loose and fragile char structure with visible cracks. NO_3_-LDH/EP and PC-LDH/EP produced more compact, porous char layers. PC-DPA-LDH/EP developed the densest and smoothest char, suggesting improved structural integrity and barrier performance, which aligns with its superior fire resistance.

The SEM and phosphorus-mapping results, shown in Figure S3 (Supplementary Information), clearly reveal differences among the composites. NO_3_-LDH/EP displays minimal phosphorus, as expected. PC-LDH/EP exhibits a uniform, dense phosphorus distribution, indicating good dispersion of the PC additive. PC-DPA-LDH/EP has a relatively high phosphorus content with localised enrichment, suggesting the strong presence of phosphorus-rich regions, which supports its enhanced flame-retardant performance.

**Figure 11:**
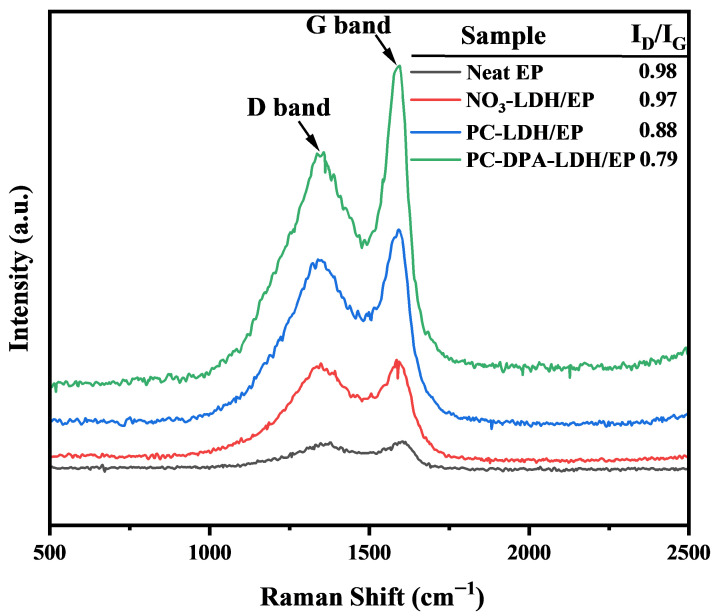
Raman spectra of the char residues of EP and its composites.

The residual chars were further analysed via Raman spectroscopy, a widely applied technique for assessing the degree of graphitisation in carbonaceous materials. The Raman spectra of the char residues from neat epoxy and its composites containing NO_3_-LDH, PC-LDH, and PC-DPA-LDH are shown in Figure 11. All samples exhibited two characteristic bands: the D band at around 1350 cm^−1^ and the G band at around 1580 cm^−1^, corresponding to disordered carbon and graphitic carbon, respectively. The intensity ratio of these two bands (I_D_/I_G_) is widely used to assess the degree of graphitisation of carbonaceous materials. The neat epoxy showed an I_D_/I_G_ value of 0.98, indicating a highly disordered char structure. With the addition of NO_3_-LDH, the ratio slightly decreases to 0.97, suggesting a minor improvement in char ordering. A more pronounced decrease is observed for the PC-LDH composite, which has a ratio of 0.88, indicating enhanced graphitic character due to the presence of phosphorus-containing groups from phosphocreatine. The PC-DPA-LDH composite has the lowest I_D_/I_G_ ratio of 0.79, reflecting the formation of a highly ordered char structure. This enhanced graphitisation is attributed to the synergistic catalytic effects of both phosphocreatine and dodecyl phosphoric acid, which facilitate the formation of a dense, thermally stable carbon residue, a crucial factor for flame retardancy.

### 3.5. Flame-Retardant Mechanisms

The flame-retardant capability of the PC-DPA hybrid epoxy/LDH nanocomposites may be attributed to an enhanced condensed-phase fire retardation mechanism, including char formation, catalytic graphitisation, and barrier effects. The PC-DPA modification improved the LDH nanoplatelet dispersion, and the LDH interlayer distance also nearly doubled (~0.8 to 1.7 nm), resulting in a well-exfoliated distribution of LDH layers in the epoxy. This homogeneous nanoscale dispersion constitutes the optimal condition for the physical barrier behaviour of LDH, as well-dispersed sheets significantly impede heat and mass transfer during combustion.

**Figure 12:**
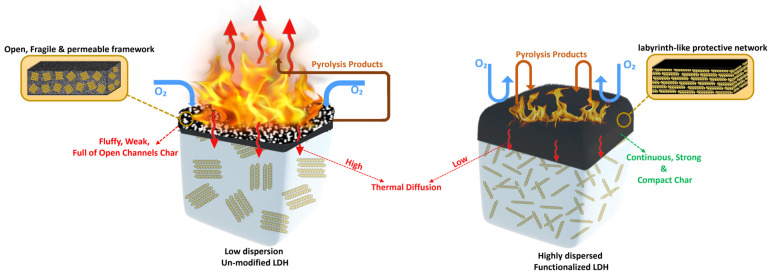
Flame-retardant mechanisms of the unmodified and modified LDH epoxy nanocomposites. Pyrolysis Products [38]: Carbon dioxide (CO_2_), Carbon monoxide (CO), Hydrocarbons (e.g., CH_4_, small alkanes/alkenes), Aromatic compounds (benzene, phenol-type species), Ethers/oxygenated organics, Water vapour (H_2_O).

Cone calorimetry demonstrated that the PC-DPA-LDH/epoxy composite has a significantly lower pHRR of about 520 kW/m^2^ which showed 46% reduction compared with  the 970 kW/m^2^ of the neat epoxy, a lower THR of 76 versus 116 MJ/m^2^, demonstrating about 34% reduction, and an about 60% reduction in the TSP (52 against 130 m^2^). These improvements are coupled with a higher char formation yield and flame resistance in flammability tests (LOI up to 29% and a UL-94 V-0 rating, whereas the neat epoxy fails the UL-94 test). These results reveal that the promotion of char by PC-DPA-LDH stems from phosphorus-rich species produced by the decomposition of PC and DPA during combustion, which form polyphosphoric acids that catalyse epoxy dehydration and carbonisation, thereby producing a protective carbonaceous char (Figure 12).

This intumescent char is much thicker and of greater volume (char expansion to ~57 mm versus 21 mm for neat epoxy), providing a strong physical barrier that protects the underlying polymer. The char containing inorganic LDH derivatives (Mg/Al oxides) is dense and continuous, with fewer cracks than the fragile neat epoxy. However, it does insulate the material well, as indicated by a lower surface flame temperature (the peak smoke temperature decreases from ~520 °C for neat epoxy to ~440 °C for PC-DPA-LDH), and impedes the release of flammable volatiles from the material via an oxygen- and heat-feedback system. Moreover, the metal species in LDH and the phosphorus/nitrogen in the hybrid modifier jointly catalyse graphitisation via the function of char. Furthermore, Raman analysis of the residues revealed a substantial reduction in the I_D_/I_G_ ratio (0.98 for neat epoxy char to 0.79 for PC-DPA-LDH), indicating an increase in graphitic structure in the char. This higher graphitic char is thermally stable and reinforces the thermal barrier. It decomposes endothermically and yields metal-oxide layers (bound water is released), which is another endothermic process that occurs in LDH and contributes significantly to thermal insulation.

Ultimately, the PC-DPA-LDH layers exfoliate to maximise these mechanisms: the nano-lamellar LDH structure can be evenly dispersed, leading to a labyrinth-like protection network throughout the char. In conclusion, PC-DPA-LDH could enhance char formation and graphitisation and simultaneously generate a compact, insulated barrier (via char and layered solids), which is highly beneficial for the flame-retardant performance of epoxy composites. All these synergistic effects of char promotion, catalytic graphitisation, physical barrier formation, thermal insulation, and efficient layer exfoliation result in the composite showing much greater resistance to ignition and flame spread over neat epoxy, as evidenced by the cone calorimetry and flammability test results.

### 3.6. Mechanical Properties

The mechanical properties of neat epoxy and its LDH-based composites are summarised in Table 4. The incorporation of unmodified NO_3_-LDH into the epoxy matrix resulted in a notable decrease in the impact strength from 16.8 kJ/m^2^ to 7.7 kJ/m^2^, indicating poor interfacial adhesion and the presence of stress concentrators. In contrast, the composite containing PC-DPA-modified LDH exhibited a comparable impact strength of 16.6 kJ/m^2^, suggesting that surface modification with phosphocreatine and dodecyl phosphoric acid effectively improved compatibility and energy dissipation at the filler–matrix interface. However, the tensile strength decreased upon the addition of both types of LDH. Neat epoxy exhibited the highest tensile strength at 62.8 MPa, whereas the PC-DPA-LDH composite showed a more pronounced reduction to 38.0 MPa, possibly due to the plasticising effect of the organic modifiers or the formation of a softer interphase.

The Young’s modulus remained nearly unchanged across the samples, indicating that the stiffness of the system was not significantly affected. Interestingly, the elongation at break increased from 3.8% for neat epoxy to 5.3% and 4.5% for the NO_3_-LDH and PC-DPA-LDH composites, respectively, reflecting enhanced deformability and toughness. These results demonstrate that functionalisation with phosphocreatine and dodecyl phosphoric acid can preserve impact performance and improve ductility, although at the cost of reduced tensile strength. The measured mechanical properties are comparable to those commonly reported for epoxy-based composites tested according to relevant ISO standards.

## 4. Conclusions

In conclusion, phosphocreatine was synthesised and used as a modifier for the green fabrication of functionalised LDHs without the use of organic solvents. A water-based PC and DPA co-modification yielded LDH with ~1.7 nm galleries and excellent dispersion in EP. As a result, PC-DPA-LDH showed a better dispersion state within the epoxy matrix than did unmodified LDH (NO_3_-LDH) and PC-LDH, which resulted in significantly improved flame retardancy. Specifically, at 7 wt% loading, PC-DPA-LDH/EP achieved a UL-94 V-0 rating and an LOI value of 29.0%, while reducing pHRR, THR, and TSP by 46.4%, 34.5%, and 59.7%, respectively, compared with neat EP, whereas NO_3_-LDH/EP and PC-LDH/EP had no rating and a V-2 rating, respectively. These improvements are attributed to the improved dispersion of PC-DPA-LDH in the epoxy matrix and the synergistic flame-retardant effects of PC and DPA.PC-DPA-LDH delivers strong flame retardancy through two coupled actions: upon heating, polyphosphoric acids form and catalyse epoxy dehydration and carbonisation, producing a dense, more graphitic intumescent char; in parallel, well-dispersed LDH platelets create an efficient heat- and mass-transfer barrier that further suppresses combustion. These findings demonstrate that the proposed co-modification strategy provides an effective and environmentally friendly route for the design of high-performance flame-retardant epoxy nanocomposites and may also offer useful guidance for the development of sustainable flame-retardant systems in other polymer matrices. In future work, further optimisation of the phosphocreatine-based modification strategy, exploration of its synergistic effects with other flame-retardant additives, and evaluation in other epoxy and polymer systems would be of considerable interest.

## Figures and Tables

**Table 1:** Thermogravimetric analysis (TGA) of LDH, organo-modified LDH, and their epoxy composites.

Sample	Residue at 800 °C (wt%)	
	Calculated	Experimental
NO_3_-LDH	--	50.9
PC-LDH	--	21.7
PC-DPA-LDH	--	19.9
Pure EP	--	13.1
NO_3_-LDH/EP	15.7	19.5
PC-LDH/EP	13.6	19.8
PC-DPA-LDH/EP	13.5	21.7

**Table 2:** LOI and UL-94 results for the EP and LDH/EP composites.

Sample	LOI (%)	UL-94	Observation
Pure EP	25.0	No rating	Fire with Sooty flame
NO_3_-LDH/EP	25.5	No rating	Fire with Sooty flame
PC-LDH/EP	26.0	V-2	Extinguished after some time
PC-DPA-LDH/EP	29.0	V-0	Extinguished immediately

**Table 3:** Combustion parameters of epoxy composites obtained from cone calorimetry tests.

Sample	TTI (s)	pHRR (kW/m^2^)	Time to pHRR (s)	FIGRA (kW/m^2^·s)	THR (MJ/m^2^)	TSP (m^2^)	Residue Mass (%)
Pure EP	75	970	190	5.1	116	130	11.1
NO_3_-LDH/EP	80	735	185	4.4	105	75.4	17.9
PC-LDH/EP	79	651	150	4.3	103	82.8	18.9
PC-DPA-LDH/EP	70	520	125	4.1	76	52.4	22.4

**Table 4:** Impact and tensile test results of the epoxy composites.

Sample	Impact Strength (kJ/m^2^)	Tensile Strength (MPa)	Young’s Modulus (GPa)	Elongation at Break (%)
EP	16.8 ± 0.6	62.8 ± 4.3	1.82 ± 0.28	3.8 ± 0.3
NO_3_-LDH/EP	7.7 ± 0.7	51.0 ± 1.5	1.72 ± 0.08	5.3 ± 0.4
PC-DPA-LDH/EP	16.6 ± 0.4	38.0 ± 2.8	1.82 ± 0.10	4.5 ± 0.3

## Data Availability

The data presented in this study are available on request from the corresponding author. The data is not publicly available because it is part of an ongoing study.

## Supplementary Materials

The following supporting information can be downloaded at: https://www.mdpi.com/article/10.3390/polym18091118/s1, Figure S1: WAXS patterns of neat EP, NO_3_-LDH/EP, PC-LDH/EP, and PC-DPA-LDH/EP nanocomposites; Figure S2: 3D FTIR spectra of pure EP, NO_3_-LDH/EP and PC-DPA-LDH/EP; Figure S3: SEM micrographs of the epoxy nanocomposites with their phosphorus elemental mapping analysis.
