# Supplementary material for: Green Fabrication of Phosphocreatine Intercalated Layered Double Hydroxides for Highly Efficient Flame-Retardant Epoxy Nanocomposites
Source: Polymers (Basel). 2026 Apr 30;18(9):1118. doi: 10.3390/polym18091118 (PMC13165554; doi:10.3390/polym18091118)
Supplement: Supplementary file 1 [file polymers-18-01118-s001.zip › polymers-4235725-supplementary.pdf]

Supplementary Information for

Green Fabrication of Phosphocreatine Intercalated Layered Double Hydroxides for Highly Efficient Flame-Retardant Epoxy Nanocomposites

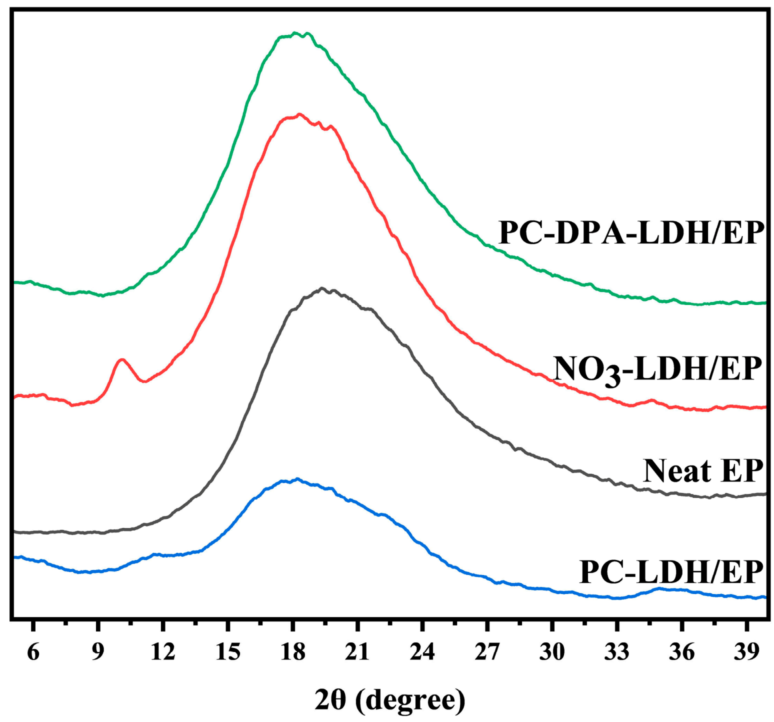

Figure S1. WAXS patterns of pure EP, NO_3-LDH/EP, PC-LDH/EP, and PC-DPA-LDH/EP nanocomposites

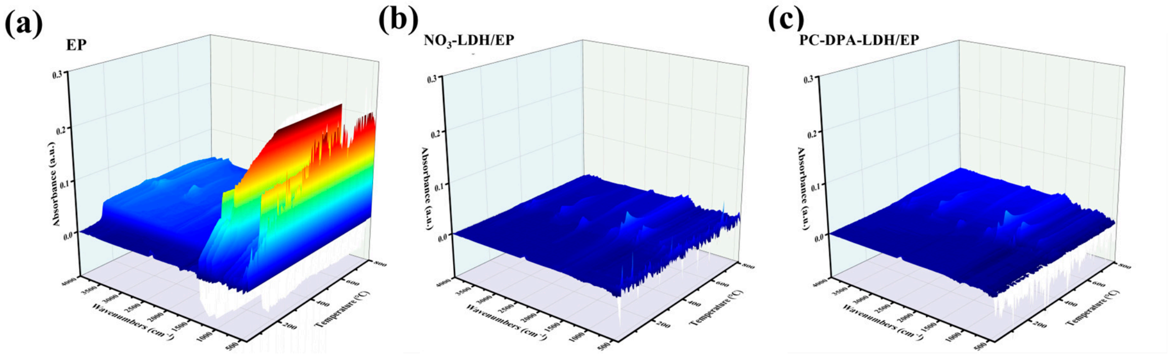

Figure S2. 3D FTIR spectra of (a) EP, (b) NO_3-LDH/EP and (c) PC-DPA-LDH/EP

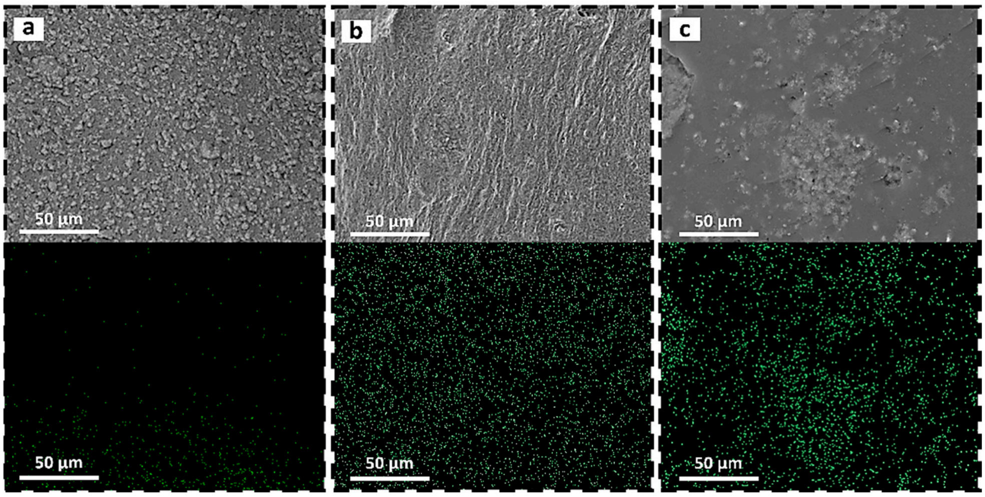

Figure S3. SEM micrographs of the epoxy nanocomposites with their phosphorus elemental mapping analysis. (a) NO_3-LDH/EP, (b) PC-LDH/EP, (c)PC-DPA-LDH/EP
